# Supplementary material for: SiC@FeZnZiF as a Bifunctional Catalyst with Catalytic Activating PMS and Photoreducing Carbon Dioxide
Source: Nanomaterials (Basel). 2023 May 18;13(10):1664. doi: 10.3390/nano13101664 (PMC10222805; doi:10.3390/nano13101664)
Supplement: Supplementary file 1 [file nanomaterials-13-01664-s001.zip › nanomaterials-2372976-supplementary.pdf]

Supplementary Materials

SiC@FeZnZiF as a Bifunctional Catalyst with Catalytic Activating PMS and Photoreducing Carbon Dioxide

Table S1. Comparison of the prepared material with other materials from the degradation literature.

Photocatalyst (Silicate and silicon based materials)	PH/Light source/Temperature	Weight (g)	C(Methylene Blue)	Time	Removal efficiency	Refs
$AgSiO_4/Ag_2CO_3$	under visible light	0.02	10ppm（50ml)	40 min	99.1%	DOI:10.1038/s41598-017-15405-6
mesoporous silicates	50°C, pH = 4	2	10ppm（50ml)	60 min	12%	doi.org/10.1016/j.jwpe.2017.07.017
6.95mmol/L H_2O_2mesoporous silicates	50°C, pH = 4	2	25ppm（50ml)	150 min	85%	DOI:10.1038/s41598-017-15405-6
0.4mlH_2O_2-40mgFtCS	40°CPH=6	0.04	100ppm (100ml)	60 min	90%	DOI: 10.1002/slct.201801945
0.4mlH_2O_2-40mgFtCS	70°CPH=6	0.04	100ppm (100ml)	30 min	100%	DOI: 10.1002/slct.201801945
0.4mlH_2O_2-40mgFtCS	80°CPH=6	0.04	100ppm (100ml)	30 min	100%	DOI: 10.1002/slct.201801945
EDAS/(TiO_2-Au)nps	450 W Xenon lamp	-	4ppm	60 min	50%	doi:10.1016/j.jhazmat.2011.12.019
Ag-/ZnO-PMOS	Tungsten bulb (200 W) PH=3	0.01	20ppm (30ml)	60 min	81%	doi.org/10.1016/j.inoche.2020.108357

TiO_2/SCS	100 W high-pressure Hg lamp	0.02	0.2 mM (40ml)	300 min	96.2%	doi.org/10.1016/j.cattod.2016.03.039
SiC nanoparticles	adsorption property	0.7	10ppm	180 min	86%	doi.org/10.1016/j.ceramint.2015.09.008
Larnite 2CaO.SiO_2	sunlight irradiation	-	-	90 min	94%	doi.org/10.1002/slct.202003046
Diopside CaO.MgO.2SiO_2	sunlight irradiation	-	-	90 min	40%	doi.org/10.1002/slct.202003046
Forsterite 2MgO.SiO_2	sunlight irradiation	-	-	90 min	42%	doi.org/10.1002/slct.202003046
Larnite 2CaO.SiO_2	UV light irradiation	-	-	90 min	97%	doi.org/10.1002/slct.202003046
Diopside CaO.MgO.2SiO_2	UV light irradiation	-	-	90 min	57%	doi.org/10.1002/slct.202003046
Forsterite 2MgO.SiO_2	UV light irradiation	-	-	90 min	46%	doi.org/10.1002/slct.202003046
C/g-C_3N_4	Adsorption–desorption equilibrium	0.1g	20ppm （100ml ）	60 min	60%	doi.org/10.1007/s11144-022-02256-0
1mM/L$H_2O_2Fe_{83}Si_5b8p_4$	electro–Fenton–like process	0.1g	20ppm (200ml)	20 min	95%	doi.org/10.1016/j.jes.2020.12.032
$Ag_6Si_2O_7/TiO_2$	sunlight irradiation -	0.1g	30ppm	20	98.6%	10.13475/j.fzxb.20200701307
(MPS-MnO_2) PH=9	sunlight irradiation	0.075	30ppm (100ml)	90 min	89.4%	doi.org/10.1016/j.matlet.2021.131367
$Te_{90}Si_5Sn_5$	sunlight irradiation	–	10ppm (100ml)	100 min	89%	doi.org/10.1016/j.vacuum.2022.110960
eSiC@ZnFeZIF	**[PMS] = 0.5 mM, pH = 6.5, T = 25 °C**	**0.050**	**20ppm (100ml)**	**60 min**	**72%**	——

Table S2. Comparison of the prepared material with other materials from the degradation literature. (Containing PMS)

Fenton-like process	PH/PMS/ Temperature	Weight	C(Methylene Blue)	Time	Removal efficiency	Refs
SiC-Fe^0	pH_{intial} = 3<br>T = 25 ℃	0. 5g/L	20 mg/L florfenicol	20 min	59.9%	DOI: 10.1016/j.seppur.2022.122187
PMS-Only	pH = 7<br>T = 25 ℃<br>[PMS] = 1 mM	0.25 g/L	20 mg/L Methomyl	60 min	85.4%	DOI: 10.1016/j.jece.2021.105358
pyrite (PyR)-PMS	pH = 7<br>T = 25 ℃<br>[PMS] = 1 mM	0.25 g/L	20 mg/L Methomyl	60 min	94.9%	DOI: 10.1016/j.jece.2021.105358
zero-valent iron (ZVI)-PMS systems	PH=7<br>T = 25 ℃<br>[PMS] = 1 mM	0.25 g/L	20 mg/L Methomyl	60 min	87.0%	DOI: 10.1016/j.jece.2021.105358
(MnO_2- PMS) system	PH=7<br>T = 25 ℃<br>[PMS] = 2 mM	0.6 g/L	20 mg/L fenuron	180 min	38%	DOI: 10.1016/j.eti.2022.102352
MFC-$MnFe_2O_4$/PMS	PH=7-8<br>T = 25 ℃	10mg cm^{-2}	100 mg/L	480 min	100%	DOI: 10.1016/j.chemosphere.2018.11.077
nanoscale-zero valent iron	PH=3<br>[PMS] = 1.25 mM<br>US power = 200 W	0.4 g/L	24mM 4-Chlorophenol(4-CP)	30 min	95%	DOI: 10.1016/j.chemosphere.2018.02.143
$CuCo_2O_4$-GO	[PMS] = 0.2 mM<br>PH=7<br>T = 25 ℃	0.1g/L	0.1 mM bisphenol A (BPA)	5 min	100%	doi.org/10.1016/j.ceramint.2015.09.008

eSiC@ZnFeZIF	**[PMS] = 0.5 mM, pH = 6.5, T = 25 °C**	**0.05g/L**	**20 mg/L (100ml)**	**60 min**	**72%**	——

Table S3. Comparison of the prepared materials with other materials in carbon dioxide reduction literature.

**Photocatalyst (CO_2 reduction)**	**PH/Light source/Temperature**	**Methane production**	**CO production**	**Refs**
Ru/$NaTaO_3$	300 W UV-enhanced Xe lamp	51.8μmol $h^{-1}g^{-1}$	139.1μmol $h^{-1}g^{-1}$	DOI:10.1038/s41598-017-15405-6
nanosized 3C-SiC	xenon lamp 25°C, pH = 7	4.9μmol $h^{-1}g^{-1}$	-	DOI:10.1021/acsami.0c19945
micro-size SiC	under visible light 25°C, pH = 7	0.76μmol $h^{-1}g^{-1}$	-	DOI: 10.1002/slct.201900102
commercial TiO2	under visible light 25°C,PH=7	1.46μmol $h^{-1}g^{-1}$	-	DOI: 10.1002/slct.201900102
ultrathin SiC nanosheets	under visible light 25°C,PH=7	3.11μmol $h^{-1}g^{-1}$	-	DOI: 10.1002/slct.201900102
SiC nanocage	25°C,PH=7 xenon lamp	-	4.68μmol $h^{-1}g^{-1}$	DOI: 10.1016/j.jcis.2022.04.111
NH2-UiO66/SiC	under visible light 25°C,PH=7	-	7.30μmol $h^{-1}g^{-1}$	DOI.org/10.1016/j.jcou.2021.101806
50 nm commercial SiC	xenon lamp 25°C, pH = 7 (2h)	0.084μmol g^{-1}	0.209μmol g^{-1}	——
eSiC@ZnFeZIF	**xenon lamp 25°C, pH = 7 (2h)**	**0.085μmol g^{-1}**	**0.509μmol g^{-1}**	——

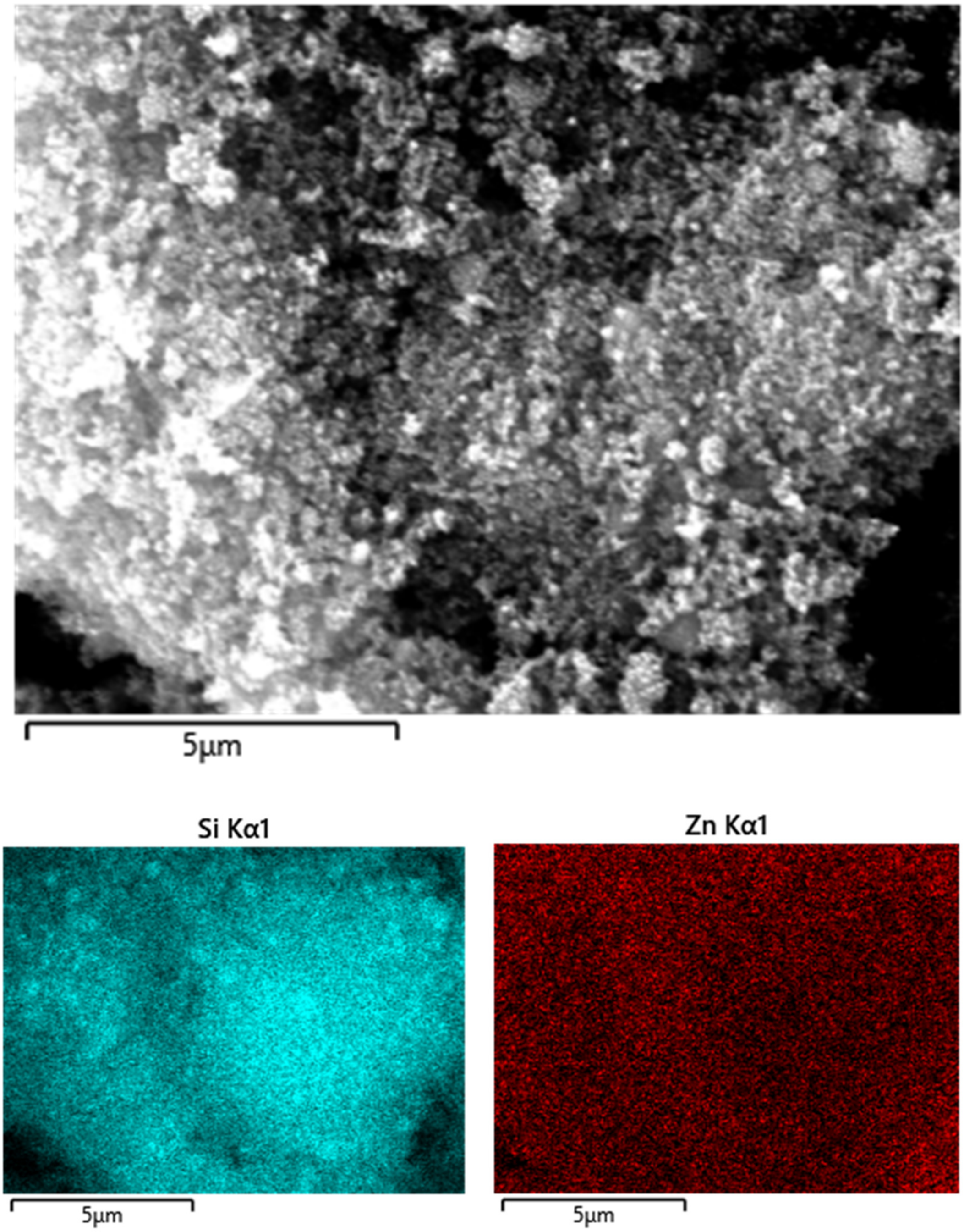
5μm
Si Kα1
Zn Kα1
5μm
5μm

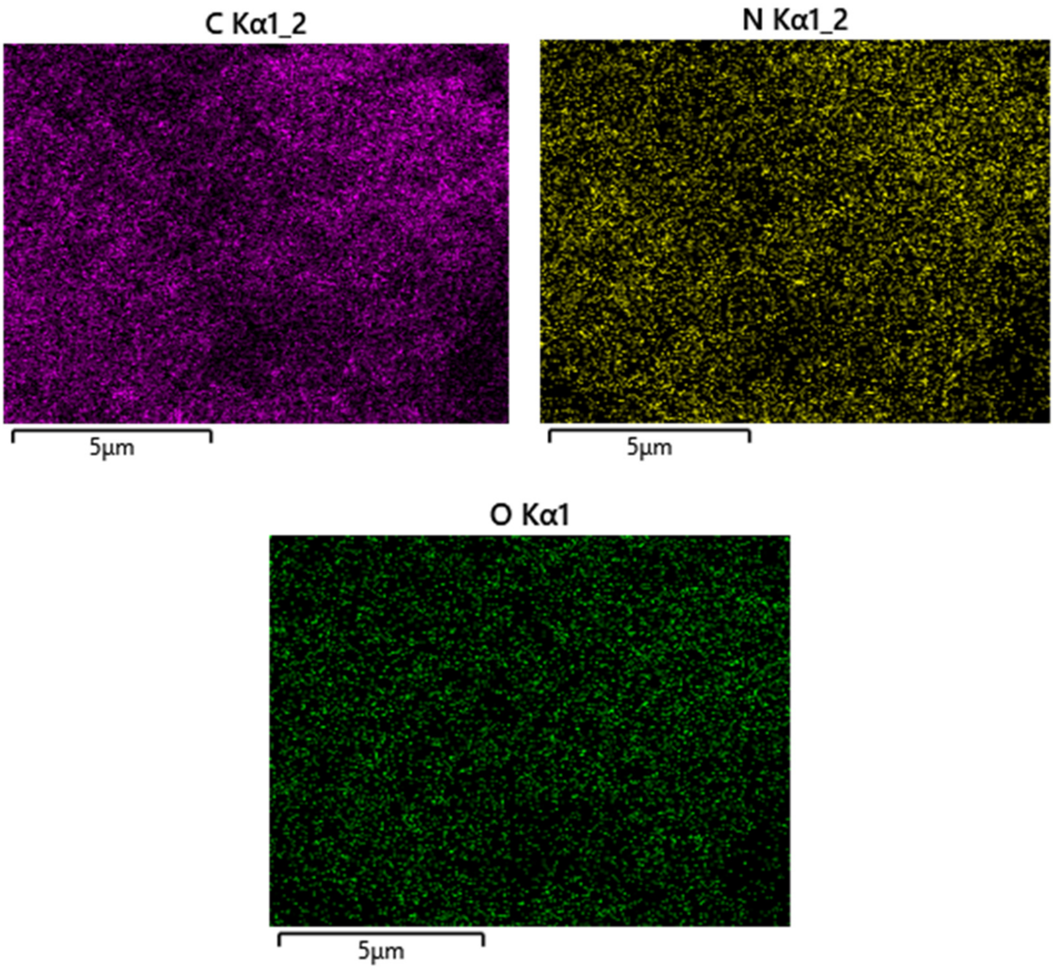

Total number spectrum of distribution map									
element	Line type	Apparent concentration	K ratio	Wt %	Wt% Sigma	Atomic %	Standard sample label	Manufacturer's standard	
C	K-line system	10.19	0.10191	42.44	0.20	61.67	C Vit	yes	
N	K-line system	10.43	0.01856	11.54	0.22	14.38	BN	yes	
O	K-line system	0.98	0.00331	1.37	0.06	1.49	SiO_2	yes	
Si	K-	49.55	0.392	29.7	0.12	18.47	SiO_2	yes	

	line system		62	1					
Zn	K-line system	21.72	0.21719	14.94	0.10	3.99	Zn	yes	
total:				100.00		100.00			

Figure S1. EDS image and element mapping of E-SiC-ZnZIF.

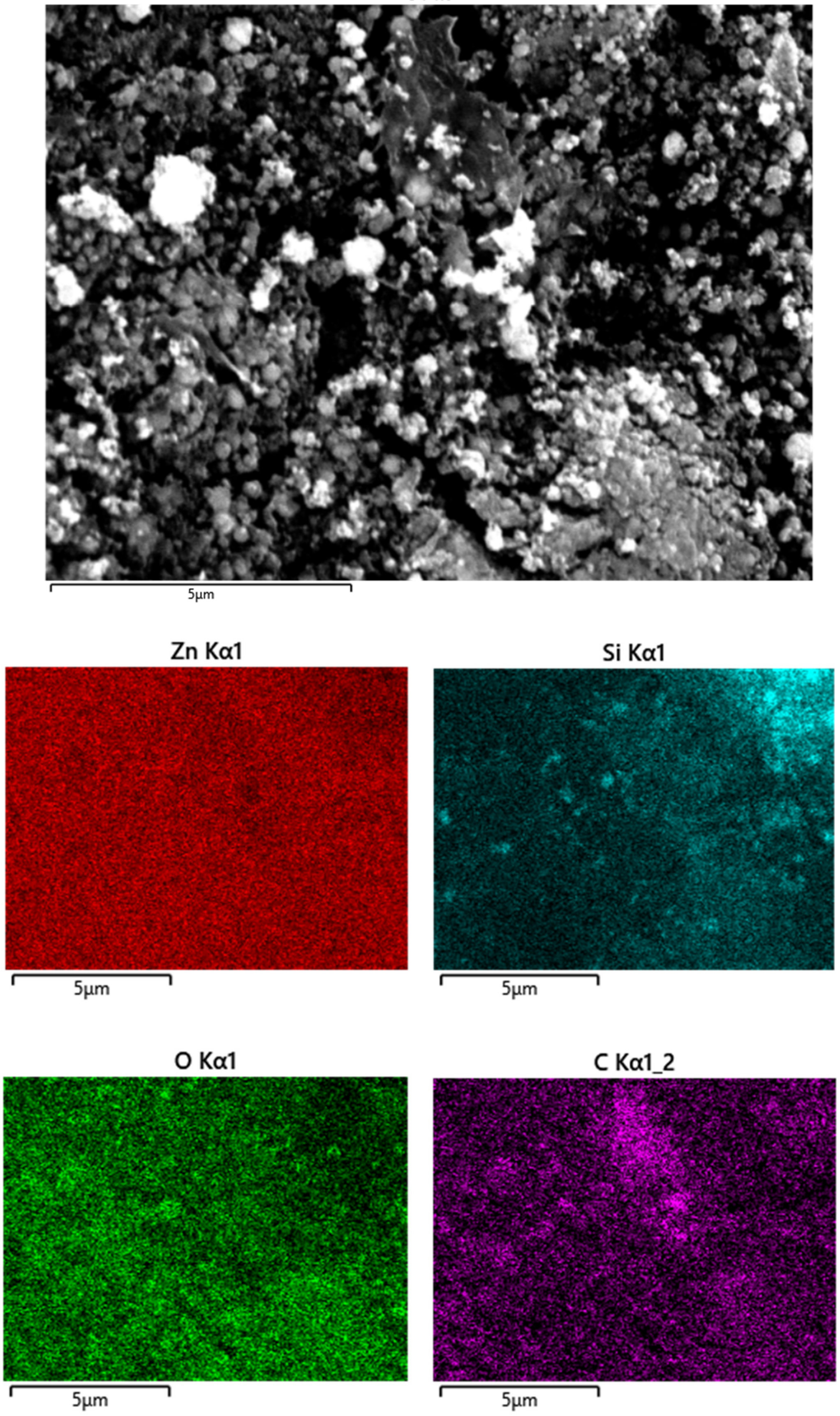
5μm
Zn Kα1
Si Kα1
5μm
5μm
O Kα1
C Kα1_2
5μm
5μm

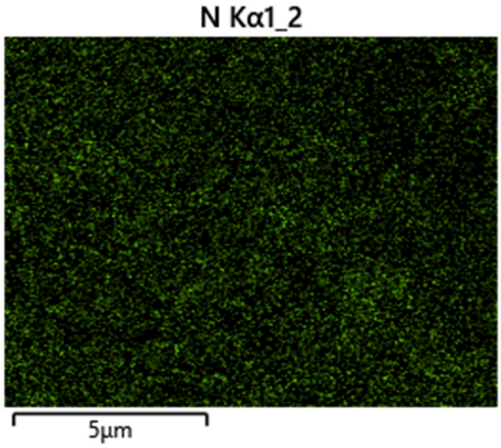

Total number spectrum of distribution map								
element	Line type	Apparent concentration	K ratio	Wt%	Wt% Sigma	Atomic %	Standard sample label	Manufacturer's standard
C	K-line system	3.39	0.03395	16.69	0.14	39.71	C Vit	yes
N	K-line system	5.27	0.00938	4.14	0.12	8.45	BN	yes
O	K-line system	10.49	0.03529	9.74	0.06	17.40	SiO2	yes
Si	K-line system	5.93	0.04701	7.02	0.03	7.15	SiO2	yes
Zn	K-line system	81.90	0.81898	62.41	0.14	27.29	Zn	yes
total:				100.00		100.00		

Figure S2. EDS image and element mapping of E-SiCZnZIF-400Ar.

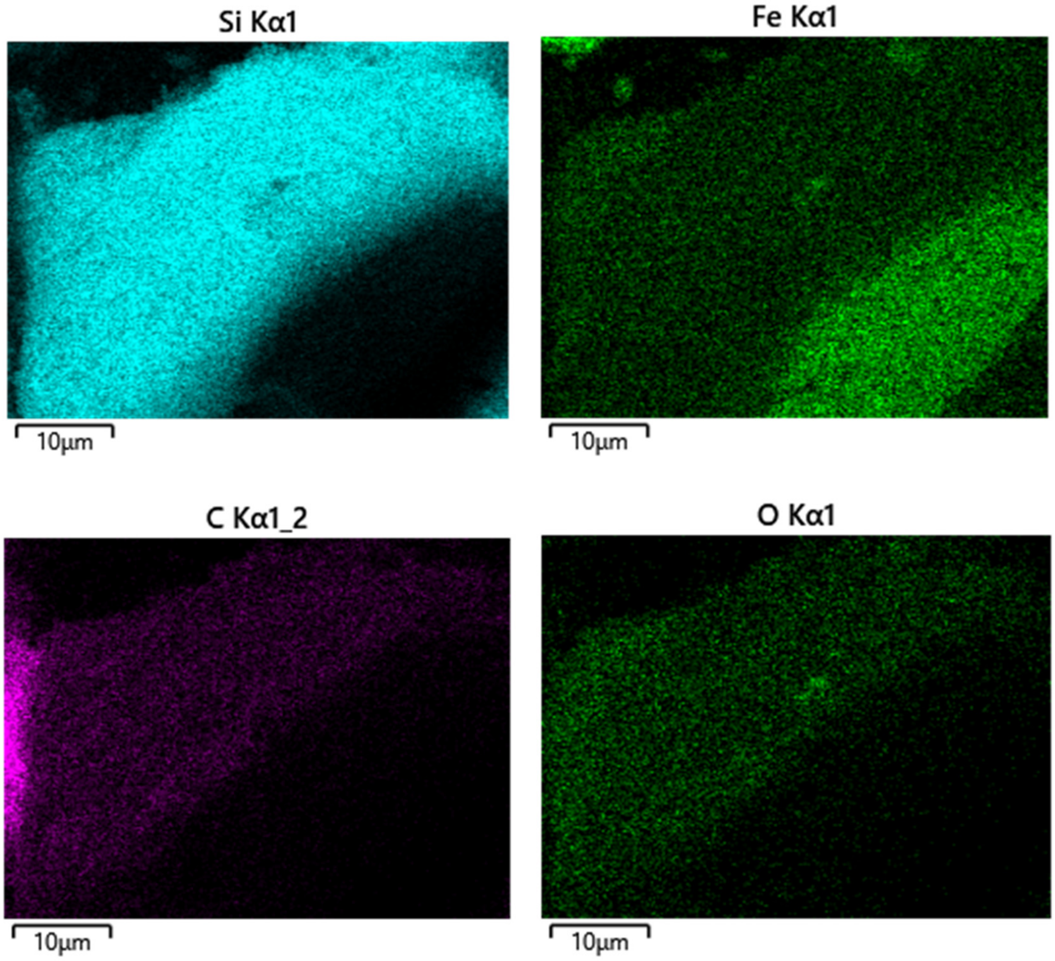

Total number spectrum of distribution map									
element	Line type	Apparent concentration	K ratio	Wt%	Wt% Sigma	Atomic %	Standard sample label	Manufacturer's standard	
C	K-line system	10.76	0.10758	47.64	0.19	68.15	C Vit	yes	
O	K-line system	5.48	0.01843	6.84	0.09	7.34	SiO2	yes	
Si	K-line system	61.65	0.48854	34.52	0.13	21.12	SiO2	yes	
Fe	K-line syste	15.91	0.15911	11.00	0.07	3.38	Fe	yes	

	m								
total<br>:				100.0<br>0		100.00			

Figure S3. EDS image and element mapping of FeZnZIF.

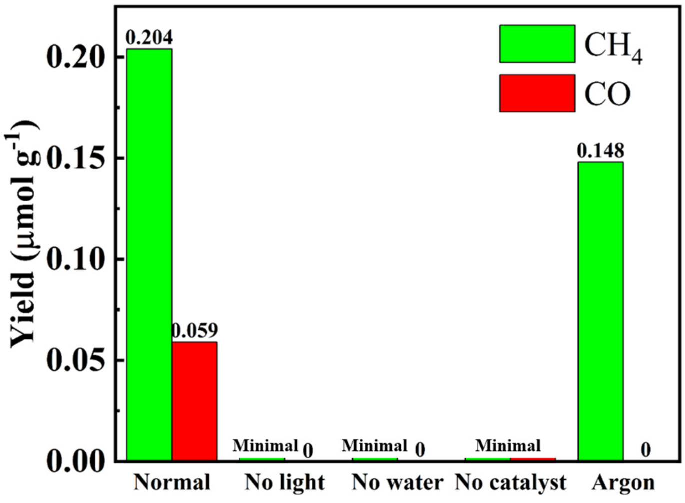

Figure S4. Photocatalytic CO_2 reduction performance under different conditions.

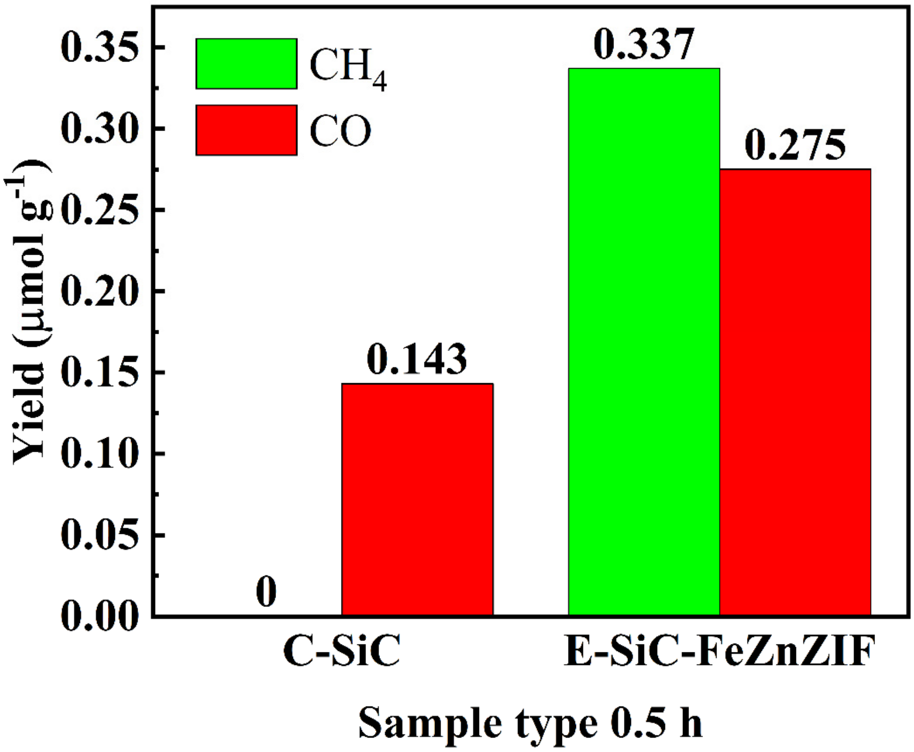

Figure S5. Photocatalytic CO_2 reduction performance by solid-gas measurement.

CO_2 reduction gas phase experiment scheme

Mix 10 mg of sample with 1.5 ml of distilled water, and then ultrasonic treatment for 10 mins to obtain a uniform slurry. Then apply it to the pre-cleaned FTO glass surface and treat it in a dry state. Then it is coated on the pre-cleaned FTO glass surface and dried under vacuum. After that, put the FTO glass at the bottom of a reactor. Place the FTO glass at the bottom of the reaction chamber (100 ml) and add 0.5 ml distilled water around the glass. Seal the reaction chamber with a quartz cover plate and treat it in vacuum for 0.5 hour, and then pass the carbon dioxide gas through a 100 mL 0.5 M glass. Pass 100 mL of 0.5 M $KHCO_3$ solution into the reaction unit to reach 80 kPa. Finally, irradiate the suspension with a 300 W xenon lamp. 300 W xenon arc lamp irradiation. Gaseous products were analyzed by gas chromatography (Techcomp GC 7). Gas chromatograph (Techcomp GC 7900). Detector (FID) and thermal conductivity detector (TCD).

Light output characteristics

Total optical power● 50 W, 19.6 W in the visible region, 2.6 W in the UV region

Spectral range● 320 to 780 nm (expandable to 2500 nm)

Compatible filters UV region, visible region, NIR region and narrow band light

Light source divergence angle● 6° on average

Spot diameter● Distance from 30 to 60 mm

Light source stability● Precision optical feedback system for direct

measurement of light output changes● Long-cycle irradiation instability ≤ ±3% (8 h)

Centralised digital power supply management control based on a micro CPU● Real-time relative irradiance display (relative value), timing function

Control modes Operating modes● Programmed mode, light-controlled mode

Current● 21 A

Lamp (consumables) life● > 1000 h (to meet the light intensity requirements of photocatalysis under normal conditions) Basic parameters Lamp power ● 300 W

Power adjustment range● 150 W to 300 W

Power ripple● 200 mVp-p (peak-to-peak)

Power supply ripple ● Digital current display

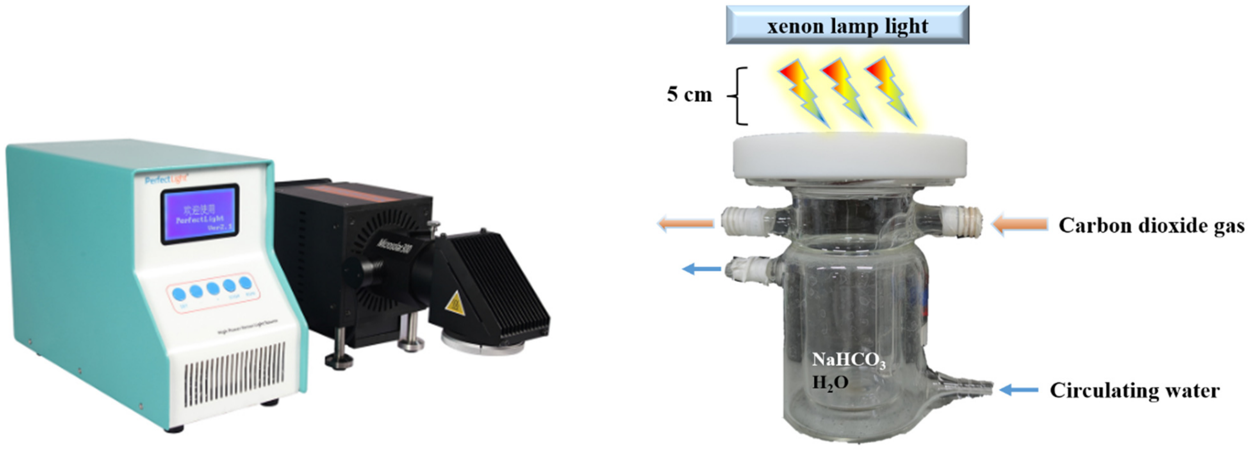

Figure S6. Light source devices and catalytic reactors

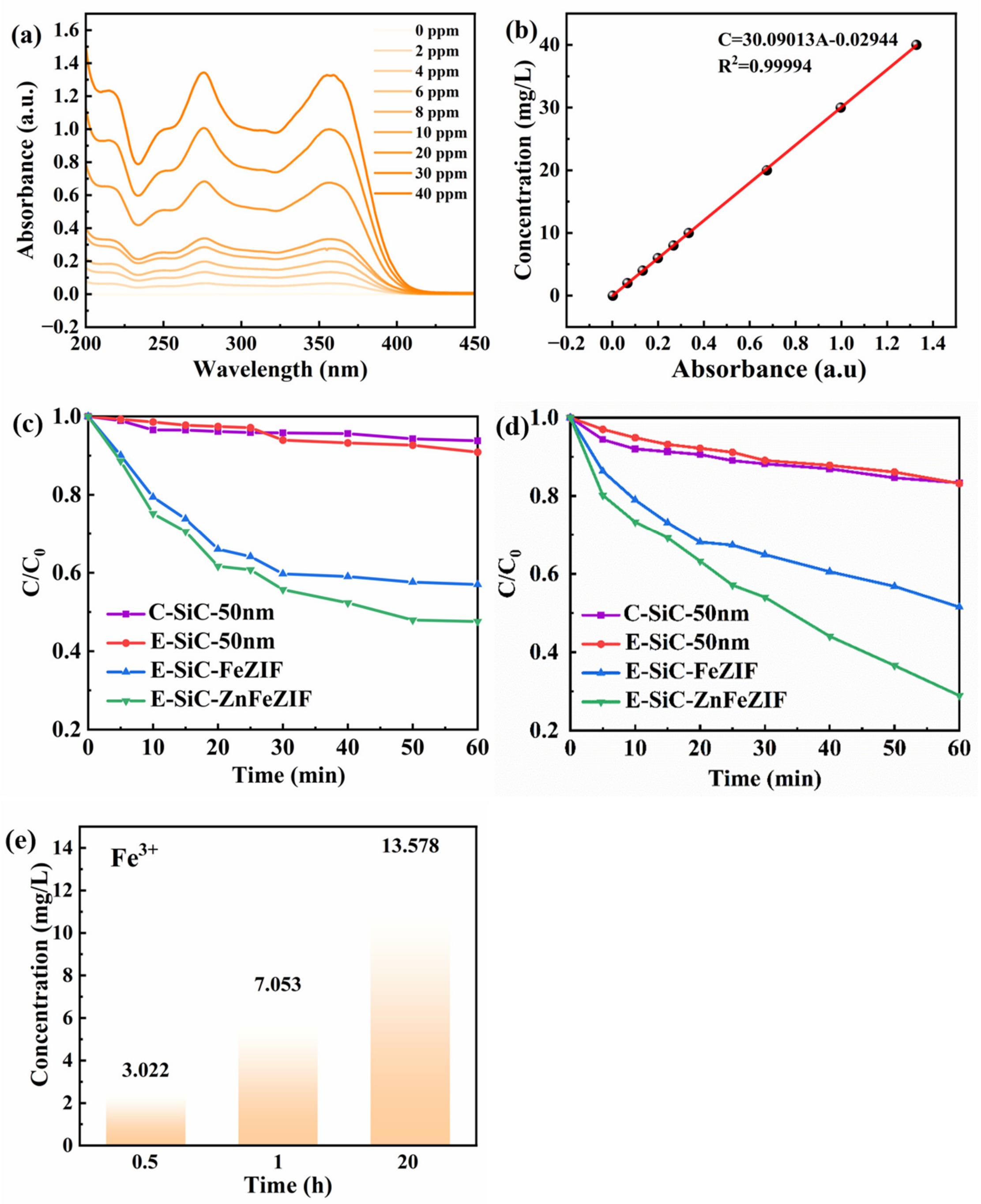

Figure. S7: The light absorption-wavelength diagram (a) and corresponding standard curve (b) of tetracycline hydrochloride at different concentrations. (c) shows the degradation of THC by various materials without agitation. (d) shows the degradation of THC by various materials under stirring at 400 rpm/min.(e) Leaching of Fe^{3+} ions in carbon dioxide reduction experiments.

Table S4. The particle size dimensions at zeta potential (pH=7).

Type	Start Date/Time	Sample ID	Eff. Diam. (nm)	Polydispersity	Baseline Index	Count Rate (kcps)	Data Retained (%)	Diffusion Coeff. (cm²/s)
DLS	2023/4/28 17:00:52	c-sic - 1	3,107.88	0.331	0.0	713.8	100.00	2.565e-09
DLS	2023/4/28 17:01:53	c-sic - 2	7,507.99	0.318	0.0	731.0	100.00	1.062e-09
DLS	2023/4/28 17:02:54	c-sic - 3	7,918.71	0.256	9.1	706.4	100.00	1.007e-09
**C-SiC**		Mean:	6,178.19	0.301	3.0	717.1	100.00	1.544e-09
		Std Err:	1,539.73	0.023	3.0	7.3	0.00	5.104e-10
		Std Dev:	2,666.89	0.040	5.3	12.6	0.00	8.841e-10

Type	Start Date/Time	Sample ID	Eff. Diam. (nm)	Polydispersity	Baseline Index	Count Rate (kcps)	Data Retained (%)	Diffusion Coeff. (cm²/s)
DLS	2023/4/28 17:12:45	etch-sic - 1	2,810.87	0.274	0.0	429.3	100.00	2.836e-09
DLS	2023/4/28 17:13:46	etch-sic - 2	2,291.75	0.029	0.0	444.3	100.00	3.478e-09
DLS	2023/4/28 17:14:47	etch-sic - 3	2,270.44	0.027	0.0	446.3	100.00	3.510e-09
**E-SiC**		Mean:	2,457.69	0.110	0.0	440.0	100.00	3.275e-09
		Std Err:	176.70	0.082	0.0	5.4	0.00	2.197e-10
		Std Dev:	306.05	0.142	0.0	9.3	0.00	3.806e-10

Type	Start Date/Time	Sample ID	Eff. Diam. (nm)	Polydispersity	Baseline Index	Count Rate (kcps)	Data Retained (%)	Diffusion Coeff. (cm²/s)
DLS	2023/4/28 17:31:09	fezif - 1	290.10	0.169	7.8	517.6	100.00	2.747e-08
DLS	2023/4/28 17:32:10	fezif - 2	285.75	0.165	7.6	503.3	100.00	2.789e-08
DLS	2023/4/28 17:33:11	fezif - 3	286.90	0.193	9.3	491.7	100.00	2.778e-08
**E-SiC-FeZIF**		Mean:	287.58	0.176	8.2	504.2	100.00	2.772e-08
		Std Err:	1.30	0.009	0.6	7.5	0.00	1.252e-10
		Std Dev:	2.26	0.015	1.0	13.0	0.00	2.168e-10

Type	Start Date/Time	Sample ID	Eff. Diam. (nm)	Polydispersity	Baseline Index	Count Rate (kcps)	Data Retained (%)	Diffusion Coeff. (cm²/s)
DLS	2023/4/28 17:45:36	znfezif - 1	323.27	0.240	2.0	480.4	100.00	2.466e-08
DLS	2023/4/28 17:46:37	znfezif - 2	319.92	0.236	5.9	476.1	100.00	2.491e-08
DLS	2023/4/28 17:47:38	znfezif - 3	322.66	0.233	2.1	476.6	100.00	2.470e-08
**E-SiC-FeZnZIF**		Mean:	321.95	0.236	3.3	477.7	100.00	2.476e-08
		Std Err:	1.03	0.002	1.3	1.3	0.00	7.932e-11
		Std Dev:	1.78	0.003	2.2	2.3	0.00	1.374e-10

Table S5. The zeta potential for each type of material (pH=7).

Type	Start Date/Time	Sample ID	Zeta Potential (mV)	Mobility (μ/s)/(V/cm)	Conductance (μS)	Sample Count Rate (kcps)	Ref. Count Rate (kcps)	RMS Residual
PALS	2023/4/26 16:42:45	32 - 1	-11.15	-0.58	800	475	3,154	2.2280e-02
PALS	2023/4/26 16:43:36	32 - 2	-12.90	-0.67	800	475	3,154	3.2387e-02
PALS	2023/4/26 16:44:28	32 - 3	-12.70	-0.66	800	475	3,154	3.8114e-02
**C-SiC**		Mean:	-12.25	-0.64	800	475	3,154	3.0927e-02
		Std Err:	0.55	0.03	0	0	0	4.6289e-03
		Std Dev:	0.96	0.05	0	0	0	8.0174e-03

Type	Start Date/Time	Sample ID	Zeta Potential (mV)	Mobility (μ/s)/(V/cm)	Conductance (μS)	Sample Count Rate (kcps)	Ref. Count Rate (kcps)	RMS Residual
PALS	2023/4/28 17:22:36	esic - 1	-4.09	-0.14	11	569	2,082	4.8290e-02
PALS	2023/4/28 17:23:28	esic - 2	-4.05	-0.14	11	569	2,082	5.3025e-02
PALS	2023/4/28 17:24:19	esic - 3	17.82	0.63	11	569	2,082	4.2588e-02
**E-SiC**		Mean:	3.23	0.11	11	569	2,082	4.7968e-02
		Std Err:	7.30	0.26	0	0	0	3.0174e-03
		Std Dev:	12.64	0.44	0	0	0	5.2263e-03

Type	Start Date/Time	Sample ID	Zeta Potential (mV)	Mobility (μ/s)/(V/cm)	Conductance (μS)	Sample Count Rate (kcps)	Ref. Count Rate (kcps)	RMS Residual
PALS	2023/4/28 17:37:18	fesic - 1	20.90	0.73	30	400	2,296	5.4627e-02
PALS	2023/4/28 17:38:09	fesic - 2	18.25	0.64	30	400	2,296	2.5063e-02
PALS	2023/4/28 17:39:00	fesic - 3	12.50	0.44	30	400	2,296	4.8366e-02
**E-SiC-FeZIF**		Mean:	17.22	0.61	30	400	2,296	4.2685e-02
		Std Err:	2.48	0.09	0	0	0	8.9947e-03
		Std Dev:	4.29	0.15	0	0	0	1.5579e-02

Type	Start Date/Time	Sample ID	Zeta Potential (mV)	Mobility (μ/s)/(V/cm)	Conductance (μS)	Sample Count Rate (kcps)	Ref. Count Rate (kcps)	RMS Residual
PALS	2023/4/28 18:01:13	znfesic - 1	24.65	0.87	71	933	2,072	4.9290e-02
PALS	2023/4/28 18:02:05	znfesic - 2	34.69	1.22	71	933	2,072	2.7312e-02
PALS	2023/4/28 18:02:56	znfesic - 3	27.64	0.97	71	933	2,072	4.3384e-02
**E-SiC-FeZnZIF**		Mean:	28.99	1.02	71	933	2,072	3.9995e-02
		Std Err:	2.98	0.10	0	0	0	6.5669e-03
		Std Dev:	5.15	0.18	0	0	0	1.1374e-02

Table S6. The BET data for each type of material.

**Samples**		commercial-nano-SiC	etched-nano-SiC	E-SiC-FeZIF	E-SiC-ZnFeZIF
BET Surface Area:		31.70 m^2/g	30.78 m^2/g	155.52 m^2/g	17.22 m^2/g
Langmuir Surface Area:		110.84 m^2/g	304.95 m^2/g	330.55 m^2/g	108.36 m^2/g
t-Plot External Surface Area:		6.91 m^2/g	28.13 m^2/g	63.37 m^2/g	14.83 m^2/g
Single point adsorption total pore volume of pores (cm^3/g)	total pore volume	0.15 cm^3/g (<2,285.6Ådiameter at P/Po=0.992)	0.27 cm^3/g (<1,772.7Å diameter at P/Po = 0.990)	0.18 cm^3/g (<1,855.0Å diameter at P/Po = 0.990)	0.079 cm^3/g (<1,663.1 Å diameter at P/Po = 0.988)
	t-Plot micropore volume	0.0096 cm^3/g	0.0011 cm^3/g	0.025 cm^3/g	0.00083 cm^3/g
BJH Desorption average pore diameter (4V/A)		345.17 Å	349.49 Å	78.34 Å	217.29 Å
Horvath-Kawazoe	Maximum pore volume at P/Po = X:	0.0126 cm^3/g X= 0.1703	0.01279 cm^3/g X= 0.1698	0.0640 cm^3/g X= 0.1690	0.00704 cm^3/g X= 0.1696
	Median pore width:	7.599 Å	7.846 Å	6.490 Å	7.832 Å
DFT Pore Size	Volume in Pores (cm^3/g)	0.00568 (<14.83 Å)	0.00148 (<14.83 Å)	0.00522 (<9.29 Å)	0.00076 (<14.83 Å)
	Total Volume in Pores (cm^3/g)	0.14910 (≤1,475.96 Å)	0.24754 (≤1,172.33 Å)	0.11465 (≤1,172.33 Å)	0.05983 (≤1,085.66 Å)
	Area in Pores (m^2/g)	0.000 (>1,475.96 Å)	6.463 (>1,172.33 Å)	17.447 (>1,172.33 Å)	5.824 (>1,085.66 Å)
	Total Area in Pores (m^2/g)	7.484 (≥14.83 Å)	18.802 (≥14.83 Å)	77.613 (≥9.29 Å)	9.815 (≥14.83 Å)
NLDFT Advanced	Volume in Pores (cm^3/g)	0.00695 (<21.14 Å)	0.00327 (<22.00 Å)	0.03799 (<14.79 Å)	0.00322 (<22.00 Å)
	Total Volume in Pores (cm^3/g)	0.13055 (≤999.93 Å)	0.24653 (≤999.93 Å)	0.16526 (≤999.93 Å)	0.07394 (≤999.93 Å)

PSD	Total Area in Pores（m^2/g）	7.878 (≥21.14 Å)	17.555 (≥22.00 Å)	35.129 (≥14.79 Å)	6.836 (≥22.00 Å)

Fig.S7 (a-b) shows the absorption-wavelength spectra of the configured standard solutions, ranging from 0-40 ppm. The final fitted linear function was C=30.09013A-0.02944 and R^2 was 0.99994. We supplemented the experiments with degradation of various catalysts by sonication for 3 min only, followed by no stirring. In the degradation experiments without stirring (Fig.S7(c)), the degradability(including adsorption) of commercially available silicon carbide and etched silicon carbide were 7% and 9%, respectively, within 60 min. The degradation efficiencies of E-SiC-FeZIF and E-SiC-ZnFeZIF were 38% and 54%. In contrast, degradation efficiencies of 18%, 18%, 50% and 72% were observed for C-SiC, E-SiC, E-SiC-FeZIF and E-SiC-ZnFeZIF, respectively, in the degradation experiments with stirring (Fig.S7(d)). Significantly lower degradation efficiencies were observed without stirring. This indicates that stirring accelerates the contact between the catalyst surface and the organic contaminants, promoting both free radical and non-free radical pathways. The effect of stirring increases the contact area and interaction between the components in the solution, facilitating the chemical reaction and speeding up the reaction rate. It allows the reactants to mix uniformly and facilitates the reaction and heat dissipation. This promotes the degradation of organic pollutants (THC).

The smaller grain size of the catalyst, the larger the specific surface area and the rougher the surface, which are more conducive to the adsorption of organic pollutants. However, if the composite grain size becomes larger, the crystal shape becomes more complete. Then the adsorption performance decreases but the degradation performance increases, the catalytic activity is dominated by the complex formed by the interaction of the composites. The adsorption properties of C-SiC and E-SiC are secondary factors influencing degradation.

The Fig. S7(e) shows the leaching concentration of Fe^{3+} ions in the carbon dioxide reduction experiment. The leaching concentrations were 3.022, 7.053 and 13.578 ppm at 0.5, 1 and 20 h. The increase in the leaching concentration of metal ions demonstrates the

destruction of the structure of the metal organic skeleton under xenon lamp irradiation. Therefore, we should further reduce the leaching of Fe ions by stabilising the structure of the composite. Tables 4-6 have been set out in the text.

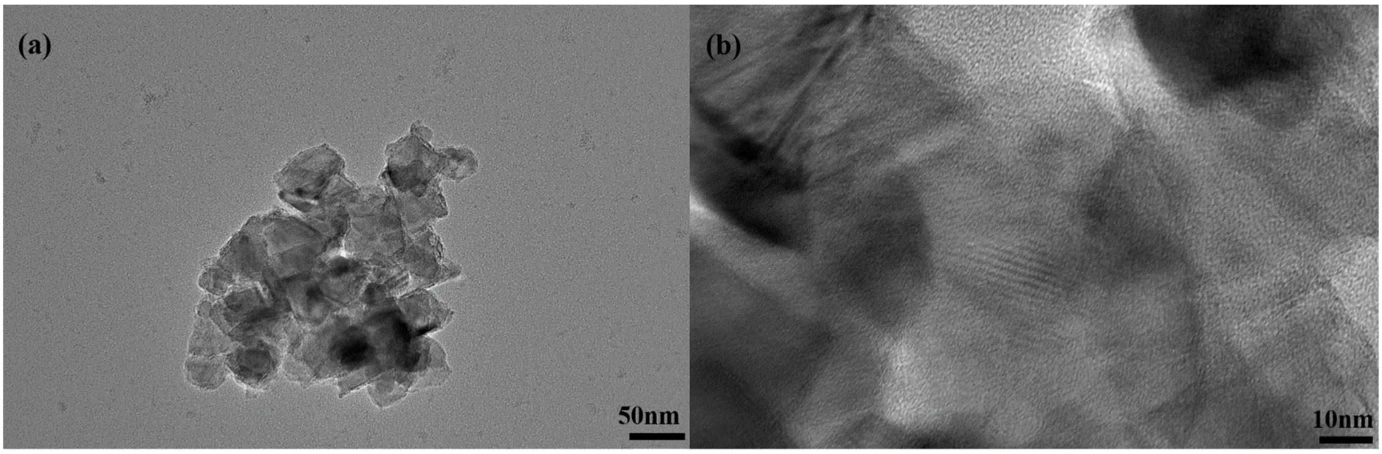

Figure. S8: TEM image of ESiC-ZnZIF and corresponding enlarged view.

Compared to the SEM image, the tiny particles observed in Fig.S8 (a) are significantly aggregated, and ZnFeZIF resembles a core-shell-wrapped modified nano silicon carbide. The lattice stripes of corresponding silicon carbide can be observed in Figure (b).

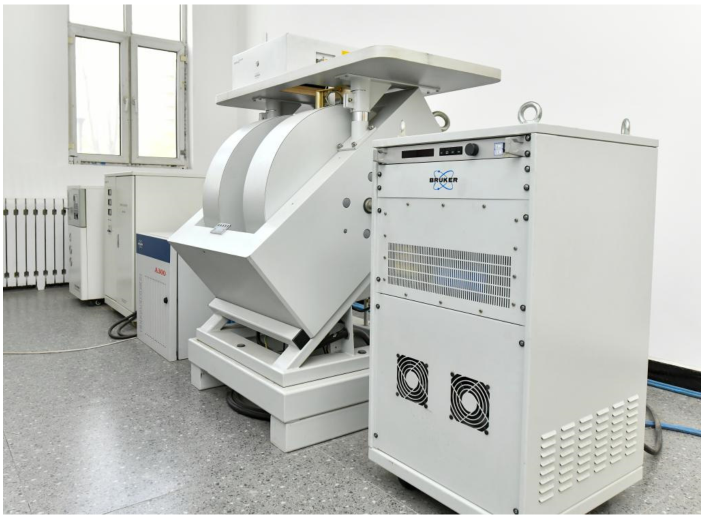

Figure. S9: Bruker A300

Test parameters

CenterField: 3507.00G Sweep Width: 100G

Power: 20mW PowerAtten: 10dB

Frequency: 9.85 GHz Modulation Amplitude: 2.00G

Modulation Frequency: 100.00 kHz

DMPO Concentration： 40mM Catalyst concentration： 0.5g/L

EPR is the premiere analytical technique for the quantitation of the number of unpaired spin spenes in a sample. As an analyocal technique Quantitative EPR offers a large dynamic range with high sensitivity for a wide range of sample types (gas, liquid, and solid). Developments in hoth instrumentation and methodology have led to increases in the precision and the accuracy of quantitative EPR measurements. For example, the Super-X bridge and the High-Sensitivity Resonator have provided significant gains in sensitivity, resulting in the ability to measure the number of unpaired spins at levels as low as 10^8 -10^9. To fully realize the potential available with such gains in instrumentation, the methods for spin quantitation are focusing on facilitating spin deter- minations and increasing the accuracy and precision of the measurements.

The CW-EPR signal amplitude A vs. magnetic field *B* (acqunrd in first-derivative mode) is influenced by several factors including sample properties and instrument settings. To avoid the unnecessary introduction of errors in the spin determination, the use of concentration or absolute spin standards is

commonly employed. This leads to the expression given by Eq. 1 for the double integral *DI* of the CW-EPR signal, computed over the field limits b_1 to b_2 containing the signal of interest.

$$\mathrm{DI}=\int_{b_1}^{b_2} dB \int_{b_1}^{B} A\,(B')dB'$$

$$=\mathrm{c}[nC_t G_R\,]\,\frac{P^{1/2}\,B_m\,Q\,n_B\,S(S+1)\,n_S}{f(B_1,B_m)} \qquad (1)$$

Thus, *DI* is proportional to the number of spins n_s, where the calibration constant c combines various spectrometer-dependent factors and the other constants and their units are:

G_R = receiver gain,
C_t = conversion time [s],
n = number of scans acquired,
P = microwave power [W],
B_1 = microwave field [G],
B_m = modulation field [G],
Q = quality factor of resonator,
n_B = Boltzmann factor for temperature dependence,
S = total elerocn spin,
n_S= number of electron spins,
$f\ (B_1,\ B_m)$ = spatial distribution of the microwave and modulation fields as experienced by the sample.

Three factors in Eq. 1 can be directly accounted for by processing of the acquired EPR signal. These factors are the receiver gain (G_R), the conversion time (C_P signal accumulation time at each field point), and the number of scans (n). By digitally correcting the measured signal, a normalized EPR spectrum is acquired which facilitates comparison of the EPR spectra acquired with different receiver settings. This normalized acquisition mode is currently implemented in the ELEXSYS line of spectrometers. The remaining terms of Eq.1 must be included explicitly for determining the number of spins n_S, or they must be accounted for by comparison with the spectrum of a known standard. Eq.1 thus offers three routes to the determination of n_S for a given sample:

(1) measurement against an amplitude reference,

(2) measurement against an standard of known concentration,

(3) calculation of n_S after determining the factor c.

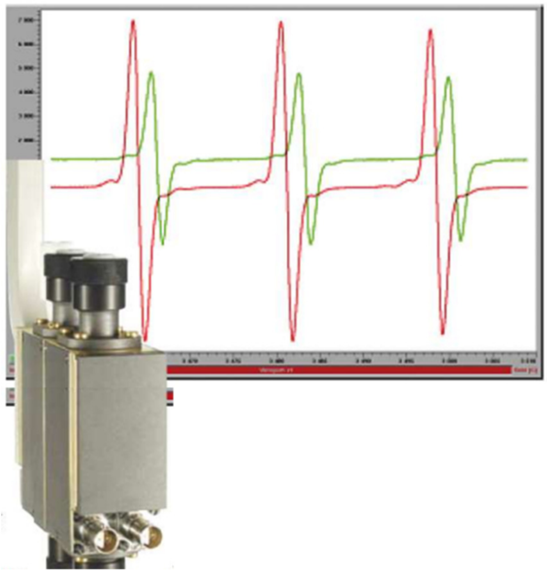

Figure S10. Tire ER 4103 DR double-cavi- resonator f left.

Typically, the EPR spectrum of the measurement is recommended to ensure that saturation or environmental effects do not adversely affect the EPR spectrum and the calculated number of spins. Typically, the EPR spectrum of the standard and the unknown are acquired sequentially in the same EPR resonator. Two sources of error in such a method are differences in Q-values and nonidentical sample positioning. To avoid such errors, the double-cavity resonator (ER4105 DR) should be used (Fig. S10). This resonator features two linked cavities which are coupled simultaneously to the microwave source so that both cavities have the same Q-value, even when subjected to different sample loading effects. For spin quantitation, one cavity is loaded with the standard while the other cavity is loaded with the unknown. This method also allows for higher throughput with increased precision,since for each unknown spectrum, a standard spectrum can be acquired under the same conditions (Fig.S10).

$$n_s^u = n_s^s \frac{DI^U B_m^S (P^S)^{1/2}}{DI^S B_m^U (P^U)^{1/2}} \quad (2)$$

where the superscripts S and U refer to the standard and unknown samples, respectively. Eq.2 is applicable for nor- malized EPR signals acquired from standard and unknown in the double cavity resonator at the same temperature and for EPR-active species with the same total electron spin S.Differences in microwave power P and field modulation amplitude B. are taken into account in Eq. 2. Note that the measurement of standard and unknown must be done under nonsaturating microwave power, and Eq. 2 also implies that the same

microwave field distribution f is experienced by both the standard and unknown samples. In the reality field within a resonator is generally not spatially uniform, and differences in sample position or volume will introduce erron into the final spin concentration calculation. A more robust method for determining the number of spins includes resonator calibration so that variability in sample size and instrument settings can be accounted for in the final calculation of the spin concentration.

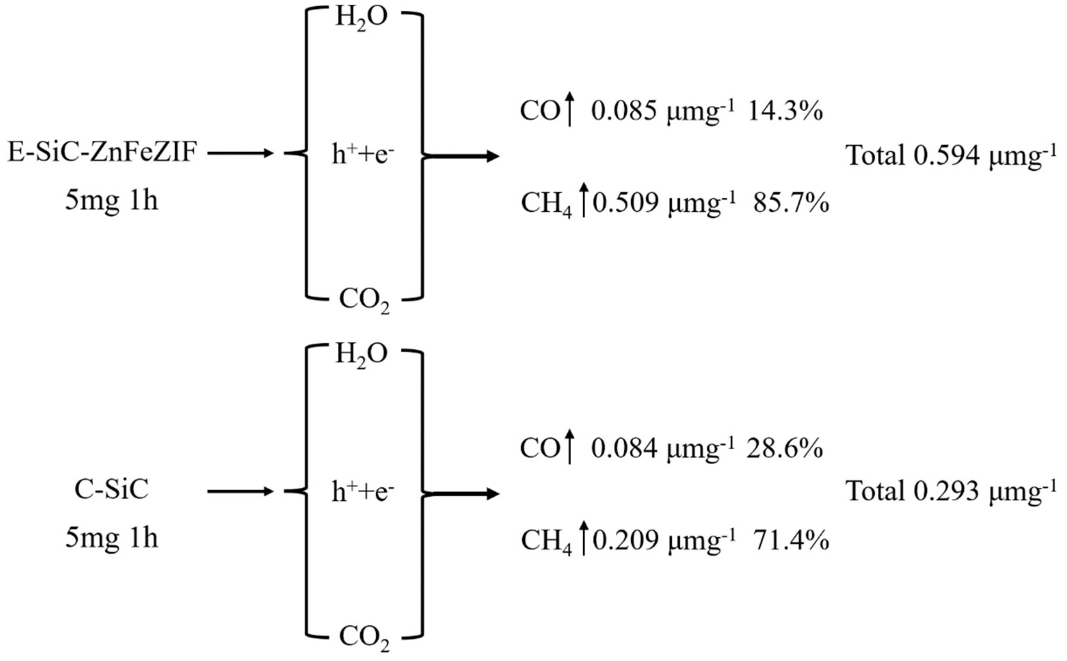

Figure S11: Conversion rates and yields of CO and CH_4 for 1 hour of material before and after compounding.
